# Genetically induced redox stress occurs in a yeast model for Roberts syndrome

**DOI:** 10.1093/g3journal/jkab426

**Published:** 2021-12-13

**Authors:** Michael G Mfarej, Robert V Skibbens

**Affiliations:** Department of Biological Sciences, Lehigh University, Bethlehem, PA 18015, USA

**Keywords:** reactive oxygen species, Eco1/ESCO2, cohesin, DNA damage, Roberts syndrome

## Abstract

Roberts syndrome (RBS) is a multispectrum developmental disorder characterized by severe limb, craniofacial, and organ abnormalities and often intellectual disabilities. The genetic basis of RBS is rooted in loss-of-function mutations in the essential *N*-acetyltransferase ESCO2 which is conserved from yeast (Eco1/Ctf7) to humans. ESCO2/Eco1 regulate many cellular processes that impact chromatin structure, chromosome transmission, gene expression, and repair of the genome. The etiology of RBS remains contentious with current models that include transcriptional dysregulation or mitotic failure. Here, we report evidence that supports an emerging model rooted in defective DNA damage responses. First, the results reveal that redox stress is elevated in both *eco1* and cohesion factor *Saccharomyces cerevisiae* mutant cells. Second, we provide evidence that Eco1 and cohesion factors are required for the repair of oxidative DNA damage such that *ECO1* and cohesin gene mutations result in reduced cell viability and hyperactivation of DNA damage checkpoints that occur in response to oxidative stress. Moreover, we show that mutation of *ECO1* is solely sufficient to induce endogenous redox stress and sensitizes mutant cells to exogenous genotoxic challenges. Remarkably, antioxidant treatment desensitizes *eco1* mutant cells to a range of DNA damaging agents, raising the possibility that modulating the cellular redox state may represent an important avenue of treatment for RBS and tumors that bear *ESCO2* mutations.

## Introduction

Roberts syndrome (RBS) (MOM #268300, MIM #269000) is a severe autosomal recessive disorder characterized by phocomelia (flipper-like appendages), microcephaly, cleft palate, syndactyly, intellectual disabilities, seizures, abnormalities in the heart, urinary, genital, and alimentary tract, spontaneous abortion, stillbirth, and possible early mortality (Van Den Berg and Francke 1993; Vega *et al.* 2005). To date, no cure exists for RBS and treatment is limited to modalities that partially improve quality of life for affected individuals (Van Den Berg and Francke 1993; Gordillo *et al.* 1993).

The sole genetic basis for RBS resides in loss of function mutations in *ESCO2*, which encodes a highly conserved (Eco1/Ctf7 in budding yeast) *N*-acetyltransferase (Skibbens *et al.* 1999; Tóth *et al.* 1999; Bellows *et al.* 2003; Hou and Zou 2005; Schüle *et al.* 2005; Gordillo *et al.* 2008). ESCO2/Eco1 acetylates a host of targets *in vivo*, most notably a DNA-binding multiprotein complex termed cohesin (Ben-Shahar *et al.* 2008; Ünal *et al.* 2008; Zhang *et al.* 2008). Cohesin is composed of structural subunits SMC1A, SMC3, and RAD21 (yeast Smc1, Smc3, and Mcd1, respectively) and auxiliary factors SA1/2 and PDS5A/B (in yeast, Scc3, and Pds5, respectively). Once acetylated, cohesins bind together DNA loci, either between sister chromatids (termed cohesion), or within a DNA molecule to generate DNA loops. Looping appears to occur through DNA extrusion, which promotes chromatin compaction and brings into registration various DNA elements (promoters, enhancers, and insulators) through which gene transcription is regulated (Kim *et al*. 2008; Rao *et al.* 2014; Banerji *et al.* 2016, 2017; Gassler *et al.* 2017; Wutz *et al.* 2017; Guo *et al.* 2018; Davidson *et al.* 2019; Kim *et al.* 2019; Golfier *et al.* 2020). ESCO2-dependent acetylation of cohesin occurs once the cohesin holocomplex is deposited onto DNA by the ATP-dependent loader NIPBL/MAU2 (yeast Scc2/Scc4) (Ciosk *et al.* 2000; Ben-Shahar *et al.* 2008; Ünal *et al.* 2008; Zhang *et al.* 2008).

ESCO2, and cohesin, function in a variety of cellular processes that include gene transcription, chromosome condensation, sister chromatid cohesion (SCC), and damage-induced cohesion (DIC). The function of the ESCO2/Eco1 family of acetyltransferases crosses all portions of the cell cycle: functioning during G1 to regulate transcription, S phase to promote SCC and chromosome condensation, and G2/M phases in which DIC promotes sister chromatid interactions during homologous recombination (HR) that occurs in response to DNA damage (Guacci *et al.* 1997; Michaelis *et al.* 1997; Losada *et al.* 1998; Skibbens *et al.* 1999; Tóth *et al.* 1999; Losada and Hirano 2001; Ivanov *et al.* 2002; Kim *et al.* 2002; Ström *et al.* 2004, 2007; Ünal *et al.* 2004, 2007; Heidinger-Pauli *et al.* 2010; Mönnich *et al.* 2011; Bose *et al.* 2012; Lu *et al.* 2014; Banerji *et al.* 2016, 2017). An early model of RBS focused on mitotic failure based on the initial characterization of Ctf7/Eco1 in yeast as a cohesion factor (Skibbens *et al.* 1999; Tóth *et al.* 1999) and findings in vertebrate cells that loss of ESCO2 function coincided with mitotic failure (stemming from SCC defects) and apoptosis, which likely precedes loss of progenitor stem cell populations (Mönnich *et al.* 2011; Morita *et al.* 2012; Whelan *et al.* 2012; Percival *et al.* 2015). However, a large body of work supports the model that RBS arises instead from transcriptional dysregulation. For instance, knockdown of ESCO2 results in zebrafish fin regeneration defects that occur independent of increased cell apoptosis (Banerji *et al.* 2016). Moreover, target genes downstream of ESCO2 (and cohesin) indeed function in bone growth and their exogenous expression suppresses phenotypes that otherwise arise from ESCO2 or cohesin (SMC3) reduction (Banerji *et al.* 2016, 2017). Early studies further linked cohesin reduction to rDNA defects (Skibbens *et al*. 2010), the first study to conceptually link cohesinopathies [RBS and Cornelia de Lange syndrome (CdLS)] to ribosomopathies (Treacher–Collins syndrome, Blackfan Anemi, Schwann–Diamond syndrome, etc.). Elegant studies document that rDNA transcription defects result in reduced ribosome biogenesis and translation (Bose *et al.* 2012; Xu *et al.* 2013; Lu *et al.* 2014). Importantly, many of the developmental defects that arise in zebrafish esco2 hypomorphs can be reduced simply through modulating translation through mTOR pathways (Xu *et al.* 2013, 2016). These findings, and others obtained from zebrafish, tissue culture cells, and tumor models (Leem *et al.* 2011; Mönnich *et al.* 2011; Xu *et al.* 2016; Guo *et al.* 2018) support a transcriptional dysregulation model of RBS. In this light, it is not surprising that CdLS, which arises through cohesin and cohesin regulator mutations, also results from transcription dysregulation and can occur in the absence of mitotic failure (Rollins *et al.* 1999; Ren *et al.* 2008; Kawauchi *et al.* 2009; Rhodes *et al.* 2010; Dorsett 2010, 2011; Mönnich *et al.* 2011; Deardorff *et al.* 2012; Dorsett and Merkenschlager 2013; Dowen *et al.* 2013; Nolen *et al.* 2013; Remeseiro *et al.* 2013; Schaaf *et al.* 2013; Skibbens *et al*. 2013; Zuin *et al.* 2014; Mannini *et al.* 2015; Yuan *et al*. 2015; Banerji *et al.* 2016, 2017; Boyle *et al*. 2017; Boudaoud *et al.* 2017; Luna-Peláez *et al.* 2019; Gudmundsson *et al*. 2019; De Koninck *et al.* 2020; Dorval *et al*. 2020). The extent to which deficits in other ESCO2 functions, beyond transcription regulation, contribute to RBS birth defects remains unknown.

ESCO2 and cohesin are both critical for DNA damage repair. While there are examples that mutation of DNA damage defense genes leads to severe birth defects (*e.g.*, Ataxia Telangiectasia, Bloom syndrome, Fanconi Anemia, Cockayne syndrome, etc.), a DNA damage model for RBS has been minimally explored. A DNA damage model of RBS is supported by findings that RBS cell lines exhibit increased sensitivity to a battery of genotoxins that include the alkylating agent mitomycin C, ionizing radiation, the topoisomerase I inhibitor camptothecin, and the topoisomerase II inhibitor etoposide (Gentner *et al.* 1985, 1986; Burns and Tomkins 1989; Van Den Berg and Francke 1993; Gordillo *et al.* 2008; van der Lelij *et al.* 2009; McKay *et al.* 2019). Moreover, altered ESCO2 and cohesin activity are tightly correlated with tumorigenesis (Zou *et al.* 1999; Oikawa *et al.* 2004, 2008; Ryu *et al.* 2007; Shepard *et al.* 2007; Barber *et al.* 2008; Solomon *et al.* 2011; Xu *et al.* 2011; Thol *et al.* 2014; Bailey *et al.* 2014; Xu *et al.* 2014; Chen *et al.* 2018; Guo *et al.* 2018; Wang and Liu 2020). Therefore, it becomes important to test whether *ESCO2* mutations, by themselves, could induce DNA damage and/or upregulate ROS pathways and thus contribute to RBS phenotypes. Here, we test the extent to which RBS can be considered, in part, a disease caused by a defective DNA damage response (DDR). We use a budding yeast model to focus in part on oxidative DNA damage induced by reactive oxygen species (ROS) produced by hydrogen peroxide (H_2_O_2_) and paraquot. In addition to prior studies that document ROS sensitivities in cells deficient in cohesin function (Ren 2005; Ren *et al.* 2008; Cukrov *et al.* 2018), this work on Eco1 provides novel insights into the role that cohesion pathways play in the defense against redox stress and highlights a novel mechanism of dysregulated endogenous ROS that contributes to reduced cell vitality in the absence of Eco1.

## Materials and methods

### Cell culture and cell cycle synchronization methods

In all experiments log phase cultures were used in an actively growing state by either growth overnight in YPD at 23°C with subculturing on day 2 followed by growth at 23°C for an additional ∼6 h. Or, cells on day 2 of growth were subcultured in YPD for overnight growth at 23°C to be used for experiments on day 3. Hydroxyurea (HU) (Sigma) was prepared at a stock concentration of 2 M in sterile water. Zeocin (Invitrogen) was added from a stock concentration at 100 mg/ml as prepared by the supplier. H_2_O_2_ (Sigma) was added from a stock concentration of either 9.8 M when preparing plates or from a 50-mM stock in sterile PBS when treating liquid cultures. Paraquat (PQ) (Sigma) was prepared at a stock concentration of 100 mM in sterile water. *N*-acetylcysteine (Sigma) was prepared at a stock concentration of 1 M in sterile 4% DMSO.

### Drug-sensitivity assays

For drop-tests, cultures were normalized to OD_600_ = 0.1 and diluted 10-fold six times in YPD. Four microliters of all seven dilutions were plated on YPD or YPD + drug and grown at the indicated temperatures for 2–4 days in the dark. Plates containing drug were prepared by adding the indicated drugs directly to the cooled agar before pouring. Plates were incubated in dark conditions to dry and used for experiments 1 day after pouring to preserve drug activity in the media.

For survival assays, cultures were normalized to OD_600_ = 1.0 in PBS. Cells were then either left untreated or treated with H_2_O_2_ for 1 h at 30°C with continuous mixing. The cells were then washed two times with PBS and serially diluted 10-fold five times. Two hundred to 250 µl of the lowest dilution were plated on YPD and survival was determined by counting colony-forming units after 2–4 days of growth at 23°C. For experiments involving *N*-acetylcysteine treatment, *N*-acetylcysteine was added to a final concentration of 25 mM to cells suspended in PBS and incubated with continuous mixing for 2 h at 23°C. Then, cells were washed in PBS two times before H_2_O_2_ treatments as described above.

### Western blot analysis

Whole cell extracts were made by TCA precipitation as previously described (Tong and Skibbens 2014). Rad53 and phosphoglycerate kinase (PGK) detection was carried out as previously described (Mfarej and Skibbens 2020).

### Quantitative growth rate and cell cycle progression analyses

For growth rate analyses, cultures were normalized to OD_600_ = 1.0 in PBS. Cells went either untreated or treated with H_2_O_2_ for 1 h at 30°C with continuous mixing. Cells were then washed two times with PBS, resuspended in YPD and diluted to OD_600_ = 0.1. Cells were incubated with continuous mixing at 23°C for 6 h with OD_600_ readings being taken at the indicated time points.

### ROS labeling analyses

Cells were normalized to OD_600_ = 1.0 in PBS and then went either untreated or treated with 1 mM H_2_O_2_ diluted in PBS for 1 h with continuous mixing at 30°C. Cells were then washed two times with PBS. 2′,7′-Dichlorofluorescein diacetate (H_2_DCFDA; ThermoFisher) was added to cells from a 1 mg/ml stock in DMSO to a final concentration of 5–10 µg/ml and incubated for 0.5–2 h at 23°C with continuous mixing. Dihydroethidium (DHE; ThermoFisher) was added to cells from 1 mg/ml stock in DMSO to a final concentration of 5 µg/ml and incubated for 2 h at 23°C with continuous mixing. Following dye incubations, cells were washed two times with PBS then used for analyses through either fluorescence microscopy or flow cytometry.

For fluorescence microscopy analysis, cells were seeded on poly-l-lysine-coated microscope slides prior to imaging [Nikon Eclipse E800, Hamatsu microscope equipped with a cooled CD (Coolsnapfx, Photometrics) and IPLab software (Scanolytics)]. All images within one biological replicate were taken using the same exposure time and a cell-sized ROI. Quantifications were performed with ImageJ by measuring the integrated density across the total surface area of each cell that was located fully in the field of view. For images where one measurement per cell could not be accurately taken due to high number of cells in the field of view, at least 100 cells were measured. For each image, 10–100 background fluorescence measurements were taken using the same cell-sized ROI and the average background intensity subtracted from the average cell fluorescence intensities to yield the normalized average cell fluorescence intensity for each image. All of the image quantifications for each sample were then averaged to obtain the average normalized fluorescence intensity for that population under the indicated treatment conditions. Fold changes in fluorescence were determined by dividing treated/mutant samples by the WT untreated sample.

For flow cytometry analyses, no dye controls consisted of an aliquot of cells from untreated samples that went for 2 h during the H_2_DCFDA incubation in PBS without labeling dye. The samples were then washed as previously described. Background levels in no dye controls were measured using the FITC channel on a BD Canto II flow cytometer with a threshold of 300, a low flow rate and counting 10,000 events. The voltage level was chosen in order to restrict the fluorescence peak to 10^2^ on the *x*-axis. All subsequent measurements in dye-treated samples were assayed using the same settings established for the no-dye controls.

## Results

### Eco1 and function are critical during redox stress

**Figure 1:**
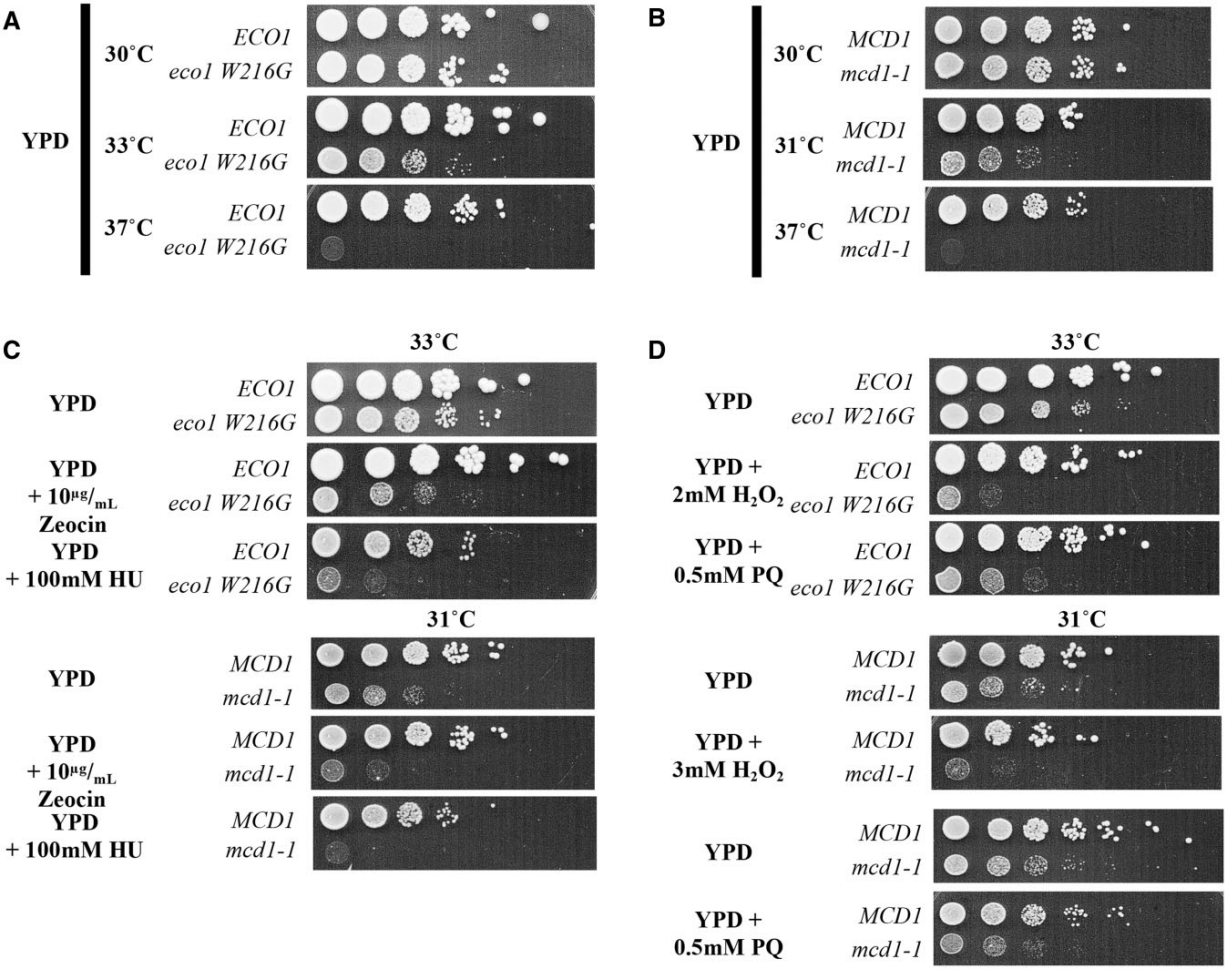
Eco1- and cohesin-dependent DNA repair pathways promote cell growth in response to redox stress; (A–C) 10-fold serial dilutions of *ECO1*, *eco1 W216G, MCD1*, and *mcd1-1* mutant strains at the indicated conditions. Cells seeded on drug-treated plates were tested at a single semipermissive temperature as indicated. Representative results shown from a total of *N* ≥ 2 biological replicates. Plates were imaged after two days of growth at the indicated temperatures.

Eco1 and cohesin function are required for DIC in response to DSBs (Ström *et al.* 2004, 2007; Ünal *et al.* 2004, 2007). The iron-responsive transcription factor Yap5, which protects against metal-catalyzed oxidation reactions, regulates *ECO1* expression in response to iron stress and DNA damage (Pimentel *et al.* 2012; Mfarej and Skibbens 2020). These findings suggest that Eco1-dependent cohesion establishment also may be important during oxidative stress. Note that *eco1 W216G is* homologous to the mutation in *ESCO2* that results in RBS in humans (Gordillo *et al.* 2008) while *RAD21* (*MCD1 in yeast*) mutation gives rise to CdLS (Deardorff 2012; Minor 2014). Both *eco1 W216G* and *mcd1-1* alleles produce cell temperature-sensitive growth and exhibit defects in the DDR (Ünal *et al.* 2007; Lu *et al.* 2010; McAleenan *et al*. 2012). We first confirmed the temperature sensitivity of *eco1 W216G* and *mcd1-1* mutant strains and then further identified an intermediate temperature (33°C for *eco1 W216G*, 31°C for *mcd1-1*) in which mutant cell growth appears sensitized (Figure  1A). All remaining experiments were performed at these experientially derived allele-sensitizing temperatures. Next, *eco1 W216G* and *mcd1-1* mutant cells, each with a matched wildtype control strain, were exposed to rich media plates or rich medium that contained either zeocin (a DSB inducer) or HU (a replication fork staller). In the absence of exogenous genotoxic agents, wildtype cells exhibit robust growth at 33°C with the *eco1 W216G* mutant strain exhibiting only moderately reduced growth at 33°C (Figure  1, A and B). *eco1 W216G* mutant cells, however, exhibited severely reduced growth on plates supplemented with zeocin and were near-inviable on plates supplemented with HU (Figure  1B). These findings greatly extend prior reports that the *eco1 W216G* allele renders cells sensitive to bleomycin and X-ray irradiation (Lu *et al.* 2010) in that HU sensitivity is unique from these genotoxic agents which induce DSBs. Additionally, while *mcd1-1* cells exhibit reduced growth at 31°C, *mcd1-1* cell growth is almost completely abolished when grown on plates supplemented with zeocin or HU (Figure  1B). Similar sensitivity to HU was observed in other *ts* cohesion mutant strains bearing the *smc3-42* mutant allele (Supplementary Figure S1A) and the *scc2-4* mutant allele (Supplementary Figure S1B).

ROS are a source of free radical electrons that cause oxidative damage to macromolecules including lipids, proteins, and DNA (reviewed in Cooke *et al.* 2003). Although oxidative DNA damage was once understood to be repaired mainly through base-excision repair (BER) and nucleotide-excision repair (NER) pathways (Gellon *et al.* 2001; reviewed in Melis *et al.* 2013), studies in both budding yeast and mammalian cell lines highlight the importance of the HR pathway in promoting genome integrity in response to ROS (Otterlei *et al.* 2000; Winn *et al.* 2003; Salmon *et al.* 2004; Yi *et al.* 2016; Choi *et al.* 2018; Novarina *et al.* 2020). Despite the importance of Eco1 in HR (Ström *et al.* 2007; Ünal *et al.* 2007; Lu *et al.* 2010; Lyons *et al.* 2013; Mfarej and Skibbens 2020a), few reports exist regarding the role of Eco1 function in ROS response pathways (Xu *et al.* 2013, 2016; Cukrov *et al.* 2018). To further test if Eco1 and cohesins co-functions are important during redox stress, we tested *eco1 W216G*, *mcd1-1*, *smc3-42*, and *scc2-4* mutant sensitivity to H_2_O_2_, which induces oxidative stress. The results reveal that *eco1 W216G*, *mcd1-1, smc3-42*, and *scc2-4* mutant cells are highly sensitive to H_2_O_2_ compared with wildtype cells (Figure  1C; Supplementary Figures S1, A and B, and S2), providing evidence for the importance of cohesion pathways in the defense against oxidative stress. In parallel, we tested *eco1 W216G* and *mcd1-1* mutant strain sensitivity to the superoxide anion inducer PQ which generates reductive stress. Interestingly, while *eco1 W216G* mutant cells exhibit severely compromised growth in the presence of PQ (Figure  1C), *mcd1-1* mutant cells are only mildly sensitive to PQ (Figure  1C). These results suggest that Eco1 function may be required for a broader range of redox stresses compared with cohesin.

### Oxidative stress hyperactivates DNA damage checkpoints in the absence of Eco1

**Figure 2:**
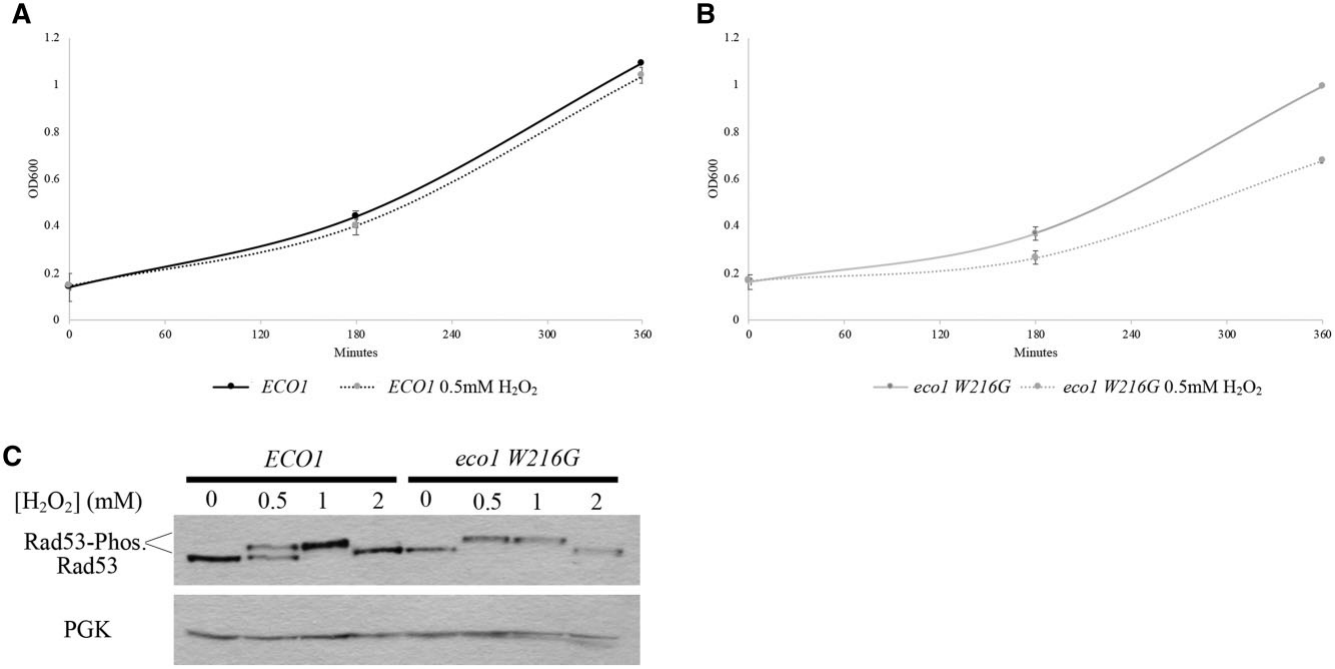
Oxidative stress results in hyperactivation of DNA damage checkpoints in an Eco1 mutant. (A, B) Growth curve of *WT* and *eco1 W216G* strains. Log-phase cells were either treated or untreated with 0.25 mM H_2_O_2_ or 0.5 mM H_2_O_2_ at 30°C for 1 h, washed and then diluted to OD = 0.1 in drug-free YPD. Cells grew for 6 h at 23°C with OD measurements taken every 3 h. *N* = 2 biological replicates. Error indicates standard error of the mean. (C) Western blot analysis of Rad53 phosphorylation in response to oxidative stress in *WT* and *eco1 W216G* strains. Log-phase cells were either treated or untreated with 0.5, 1, or 2 mM H_2_O_2_ at 30°C for 1 h prior to sample harvesting and subsequent western blot. PGK is used as a loading control.

If oxidative stress induces DNA damage, then the slowed growth rate exhibited by *eco1* mutant cells might be due to hyperactivation of DNA damage checkpoints. To test this prediction, log-phase *WT* and *eco1 W216G* cells were exposed to 0.25 mM H_2_O_2_ or 0.5 mM H_2_O_2_ for 1 h at 30°C, washed and resuspended in drug-free media. Cell density (OD_600_) was then measured over a 6-h time course. The results show that *eco1 W216G* cells exhibit approximately a 35% reduction in growth rate when exposed to medium containing 0.5 mM H_2_O_2_, relative to the growth rate of *ECO1* cells (Figure  2, A and B). We reasoned that the reduced growth rate of *eco1 W216G* cells, in the presence of 0.5 mM H_2_O_2_, may result from hyperactivation of a DNA damage checkpoint. To test this possibility, we assessed the phosphorylation state of Rad53, which becomes phosphorylated in response to DNA damage (Allen *et al.* 1994; Sun *et al.* 1998). In the absence of H_2_O_2_, Rad53 remained unphosphorylated in both wildtype and *eco1 W216G* mutant cells. As expected, wildtype cells exposed to 0.5 mM H_2_O_2_ contained phosphorylated Rad53, but this level of H_2_O_2_ was only sufficient to induce roughly 50% of Rad53 phosphorylation. In contrast, all of Rad53 appeared phosphorylated in *eco1 W216G* mutant cells in response to 0.5 mM H_2_O_2_ (Figure  2C).

### *ECO1* mutation elevates endogenous ROS levels

**Figure 3:**
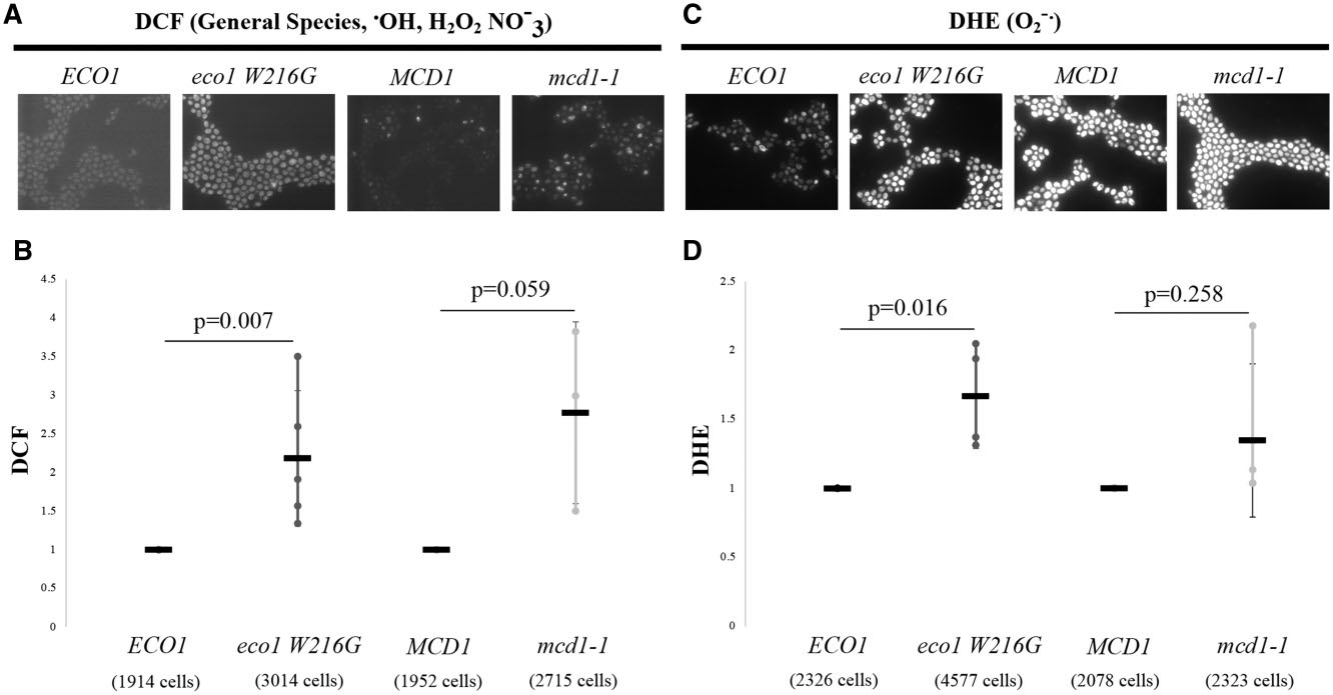
Eco1 and cohesin mutation causes endogenous ROS overproduction. (A and C) Fluorescence micrographs of *ECO1, eco1 W216G, MCD1*, and *mcd1-1* strains following incubation in either H_2_DCFDA or DHE for 2 h at 23°C prior to imaging. (B and D) Quantifications of the data in (A and C), respectively. Biological replicates *N* ≥ 4. Error bars indicate standard deviation. Statistical analysis was performed using one-tailed, Student’s *T*-test (α ≤ 0.05) for H_2_DCFDA staining and two-tailed Student’s *T*-test (α ≤ 0.05) for DHE staining.

DNA damage upregulates endogenous ROS pathways which in turn are capable of further damaging DNA and other cellular macromolecules (Evert *et al.* 2004; Rowe *et al.* 2008; Kang *et al.* 2012; Rowe *et al.* 2012; Marullo *et al.* 2013; Yi *et al.* 2016; Choi *et al.* 2018). Given *eco1 W216G* mutant cell hypersensitivity to ROS and genotoxic agents, we wondered whether cells with reduced Eco1 function might exhibit increased ROS—even in the absence of exogenous stresses. To test this hypothesis, we quantified ROS levels in *eco1 W216G* and *mcd1-1* mutant cells. Log phase *eco1 W216G* and *mcd1-1* mutant strains, and matched wildtype strains, were treated with 1 mM H_2_O_2_ for 1 h at 30°C, washed and then incubated in the presence of H_2_DCFDA. H_2_DCDFA is a cell permeable nonfluorescent reagent, that upon oxidation by ROS and reactive nitrogen species, fluoresces and becomes trapped in the cell (reviewed in Kalyanaraman *et al.* 2012). Importantly, *eco1 W216G* mutant cells produced an H_2_DCFDA signal over two times greater than matched wildtype cells, in the absence of H_2_O_2_-induced stress when ROS levels were analyzed by fluorescence microscopy (Figure  3, A and B; Supplementary Figure S3A). Similarly, increased H_2_DCFDA fluorescence levels in *eco1 W216G* cells were also detected when measured by flow cytometry (Supplementary Figure S4, A–C). *mcd1-1* mutant cells exhibited over a 2.5-fold increase in H_2_DCFDA signal, compared with matched control wildtype cells, in the absence of H_2_O_2_-induced stress although this increase was below the threshold of statistical significance (Figure  3, A and B; Supplementary Figure S3A). Thus, reduction in Eco1/cohesin pathways are solely sufficient to upregulate endogenous levels of ROS, consistent with prior findings (Xu *et al.* 2013; Cukrov *et al.* 2018). Given that *eco1* and *mcd1* mutant cells induce ROS in the absence of exogenous stress, it became important to test the ability to increase ROS production upon H_2_O_2_ exposure.

Neither *eco1 W216G* or *mcd1-1* mutant cells, however, increased ROS levels in response to H_2_O_2_ treatment beyond that induced by the mutation alone (Supplementary Figures S4, A–C and S5, A–C). This is likely due to ROS levels that remain higher in *eco1 W216G* and *mcd1-1*, with or without H_2_O_2_, than respective wildtype strains exposed to H_2_O_2_ (Figure  3, A and B; Supplementary Figure S5, A and B).

H_2_DCDFA does not react with superoxide anions (reviewed in Kalyanaraman *et al.* 2012). Thus, we repeated the above experiments, but this time incubating cells with DHE, which specifically reacts with superoxide anions to produce a fluorescent and cell-impermeable marker (reviewed in Kalyanaraman *et al.* 2012). Results obtained from DHE in untreated cells were overall consistent with those obtained using H_2_DCFDA. For instance, *eco1-W216G* exhibited increased superoxide anion levels, relative to matched controls, in the absence of H_2_O_2_-induced stress (Figure  3, C and D; Supplementary Figure S4B). *mcd1-1* mutant cells also exhibited an increase DHE intensity, but below the threshold of statistical significance (Figure  3, C and D; Supplementary Figure S4B). DHE staining, in response to H_2_O_2_ treatment, also revealed notable differences from H_2_DCFDA results. For instance, the DHE signal was not significantly different between *ECO1* and *eco1 W216G* in the presence of H_2_O_2_-induced stress (Supplementary Figure S5, D–F). *eco1* mutant cells in fact appear to have slightly diminished fluorescent intensity, although this difference was not statistically significant from *ECO1* wildtype samples (Supplementary Figure S5, D–F). Moreover, DHE signal in the *MCD1* or *mcd1-1* strains did not change in response to H_2_O_2_ stress although DHE fluorescence levels remained higher in the *mcd1-1* relative to wildtype controls in response to H_2_O_2_ (Supplementary Figure S5, D–F).

### ROS contribute to genotoxicity in the absence of Eco1

**Figure 4:**
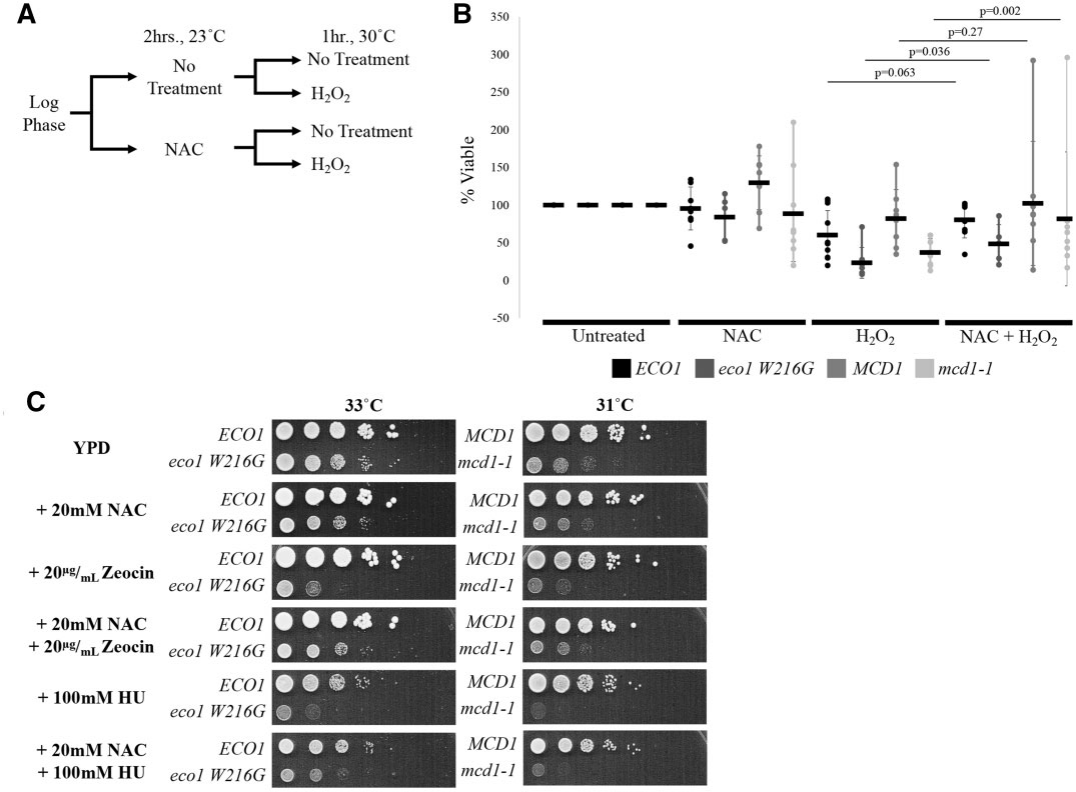
Rescue of Eco1 and cohesin mutation-associated defects with antioxidant treatment. (A) Quantification of *ECO1*, *eco1W216G*, *MCD1*, and *mcd1-1* strain viability at 2–4 days following exposures to either no drug or 25 mM NAC at 23°C for 2 h followed by subsequent incubations at 30°C for 1 h either with or without 1 mM H_2_O_2_. Percent viability was determined by averaging the ratios of viability (drug-treated colonies/untreated colonies) across at least *N* ≥ 4 biological replicates. Error bars indicate standard deviation. Statistical analysis was performed using one-tailed, Student’s *T*-test (α ≤ 0.05); (B and C) 10-fold serial dilutions of *ECO1*, *eco1-W216G, MCD1*, and *mcd1-1*. Each yeast strain was grown for 2 days at the indicated temperatures on YPD or YPD supplemented with either 25 mM NAC, 10 µg/ml zeocin, 100 mM HU, NAC + zeocin, or NAC + HU.

Our findings that mutation of either *ECO1* or *MCD1* is solely sufficient to increase endogenous ROS levels, in the absence of exogenous oxidative stress, suggests strategies through which mutant cell phenotypes could be ameliorated. To test this, we used the ROS-scavenging antioxidant *N*-acetyl cysteine (NAC) (Aruoma *et al.* 1989; Carter *et al.* 2005; Reliene *et al.* 2009; Tang *et al.* 2015). Log-phase *ECO1, eco1 W216G, MCD1*, and *mcd1-1* mutant cell cultures were split with half untreated and the other half pretreated with 25 mM NAC for 2 h at 23°C. Cells were then washed, resuspended in PBS and either remained untreated or exposed to 1 mM H_2_O_2_ at 30°C for an additional 1 h (Figure  4A). The resulting cultures were then plated onto fresh medium, maintained at 23°C and viability assessed 48–96 h later. All viability data are normalized to the untreated cultures.

We reasoned that, if NAC neutralizes exogenous H_2_O_2_ that generates oxidative stress, then cells pretreated with NAC should reduce the genetically induced ROS load and promote increased cell viability after subsequent oxidative stress. Following a 1-h treatment with 1 mM H_2_O_2_, *ECO1, eco1 W216G*, *MCD1*, and *mcd1-1* cells exhibited reductions (roughly 40%, 75%, 30%, and 65%) in cell viability (Figure  4A). When pretreated with NAC, however, both *eco1 W216G* and *mcd1-1* cells exhibited a significant increase in cell viability (Figure  4A). While matched wildtype controls appeared to benefit from NAC pretreatment, the results were not statistically significantly which highlights the effects of reduced cohesin function in promoting ROS production (Figure  4A).

The increase in mutant cell viability, when pretreated with NAC and subsequently followed by a short exposure to H_2_O_2_, suggested that increased endogenous ROS levels might play a key role in *eco1 W216G* and *mcd1-1* decreased resistance to DNA damage. To formally test whether reducing ROS levels through NAC would protect cells against exogenous DNA damage, we supplemented rich medium with HU or zeocin as well as NAC. Note that these genotoxic agents produce DNA damage defects that do not directly rely on ROS. As before, the results show that both *eco1 W216G* and *mcd1-1* cells exhibit hypersensitivity to HU and zeocin (Figure  4, B and C). When medium is supplemented with NAC, in addition to either zeocin or HU, *eco1 W216G* mutant cells exhibited reduced genotoxic sensitivity and a partial rescue of growth defects (Figure  4B). NAC supplementation similarly provided a growth benefit to *mcd1-1* mutant cells simultaneously exposed to zeocin. This protection, however, did not extend to *mcd1-1* mutant cells concurrently exposed to NAC and HU (Figure  4C).

## Discussion

Oxidative stress is implicated in aging and numerous human maladies that include neurodegeneration, heart disease, and cancer (reviews: Finkel and Holbrook 2000; Cooke *et al.* 2003; Barnham *et al.* 2004; Valko *et al.* 2006). Oxidative reactive species damage proteins, lipids, and produce mutagenic DNA adducts such as modified bases, apurinic/apyrimidinic sites, single strand breaks (SSBs), and DSBs (reviewed in Kryston *et al.* 2011). NER repair and BER are considered primary defenses against oxidative DNA damage. The first major revelation of the current study is that Eco1 and cohesin function as physiologically relevant defenses against oxidative stress. Our studies extend prior work that demonstrated functional overlap between HR and oxidative stress regulators (Yi *et al.* 2016; Choi *et al.* 2018; Novarina *et al.* 2020). The current study thus provides strong support for a model that Eco1/cohesin are important regulators of HR during oxidative stress (Xu *et al.* 2013, 2016; Cukrov *et al.* 2018). Given the variety of mutagenic modifications induced by oxidative DNA damage, one limitation of this study is that the specific types of oxidative DNA damage repair that Eco1 and cohesin promote remain unknown. It is known, however, that DIC plays a critical role during DSB repair. Future studies may provide important insights as to the physiological contributions that Eco1/cohesin provide to repair other oxidative lesions (*e.g.*, base modifications, SSBs, etc.). While DIC is likely one pathway through which Eco1/cohesin resolve oxidative stress, we further note that Eco1/cohesin are critical regulators of gene transcription and that this parallel pathway may be, in parallel, important in oxidative stress responses.

**Figure 5:**
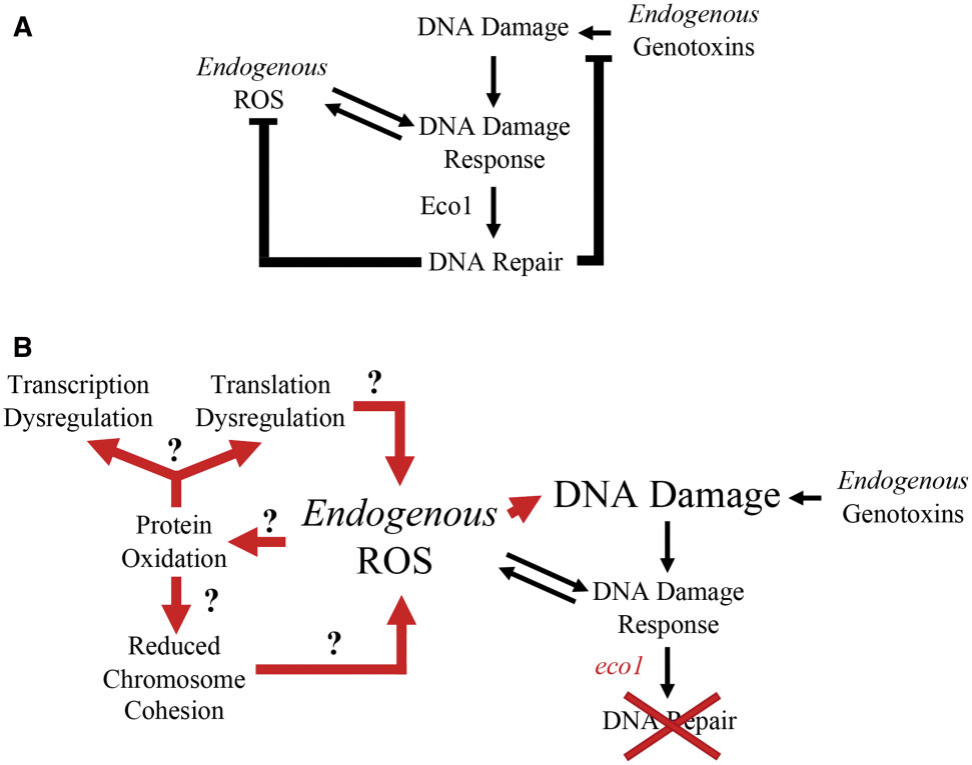
A model for the role of Eco1 in the defense against ROS-induced stress. (A) Eco1 function supports DNA repair which is sufficient to defend against sources of genotoxicity and in turn regulates endogenous ROS levels. (B) In the absence of Eco1 function, compromised DNA repair functions lead to an accumulation of DNA damage and increased ROS. ROS dysregulation contributes in a feed-forward circuit to further increase DNA damage levels. ROS may also result in global protein oxidation and perhaps enhance transcriptional, translational and chromosome cohesion defects associated with Eco1 mutation.

What is the mechanism of Eco1/cohesin sensitivity to DNA damage? DIC is required for bringing sister chromatids into close physical proximity to promote strand invasion reactions during high fidelity HR (Birkenbihl and Subramani 1992; Kadyk and Hartwell 1992; Sjögren and Nasmyth 2001; Ström *et al.* 2004, 2007; Ünal *et al.* 2004, 2007). DNA damage, however, also induces ROS upregulation, providing a feedback mechanism that activates repair processes. The second major revelation of the current work is that Eco1/cohesin regulates endogenous ROS levels during DNA damage. ROS are upregulated in the cell in response to a variety of stresses, including DNA damage, which functions to post-translationally modify DDR regulators like Yap1 in budding yeast and ATM in mammals through cysteine side chain oxidation (Moye-Rowley *et al.* 1989; Coleman *et al.* 1999; Delaunay *et al.* 2000; Delaunay *et al.* 2002; reviewed in Rodrigues-Pousada *et al.* 2004; Guo *et al.* 2010). In cells with functional DIC, endogenous sources of DNA damage are repaired through NER, BER, and HR pathways—each of which keeps ROS levels in check (Figure  5A). In the absence of DIC via *ECO1* or cohesin mutation, ROS are upregulated and background levels of oxidative stress produce increased genotoxic stress (Figure  5B). In combination with other studies that implicate deficiencies in Esco2 or cohesins in driving elevated ROS levels (Xu *et al.* 2013, 2016; Cukrov *et al.* 2018), our findings raise important implications regarding the etiology of RBS. For example, elevated ROS levels present in cells deficient in Esco2 and cohesin functions are likely to incur additional levels of macromolecular damage to DNA, proteins, and lipids. This model suggests that RBS cytotoxicity may arise through several synergistic ROS-dependent mechanisms that include oxidation of DNA and proteins that may contribute to defects in transcription, translation, and SCC (Figure  5B) (reviewed in Mfarej and Skibbens 2020b).

The molecular description of Eco1 function is likely still in its infancy. For instance, Eco1 not only acetylates Smc3 (SCC establishment), but also Mcd1 (DIC), Mps3 (nuclear organization), and the inner surface of the PCNA sliding clamp that regulates repair polymerase processivity during HR (Antoniacci *et al.* 2004; Heidinger-Pauli *et al.* 2009; Billon *et al*. 2017). The final revelation of the current study is that Eco1 and cohesin exhibit different redox stress characteristics. For example, both *eco1* and *mcd1* mutants are sensitive to H_2_O_2_-induced stress. Between the two, however, strains lacking full Eco1 function appear more sensitive to the superoxide anion generator PQ. This suggests that Eco1-dependent pathways function in response to a wider range of redox stresses than cohesin-dependent pathways and may reflect a broader role for Eco1 in the maintenance of genomic integrity. Similarly, while both *eco1* and *mcd1* mutant strains exhibit increased endogenous levels of oxidative ROS, *eco1* mutant cells exhibit higher levels of superoxide anion in the absence of challenges whereas the *mcd1* mutant exhibits higher superoxide anion levels in response to H_2_O_2_-induced stress. The effects of superoxide anion levels in *eco1* and *mcd1* mutants is unclear given data that superoxide anion serves as a cell death inducer in response to endogenous DNA damage and UV-C treatment (Rowe *et al.* 2008, 2012; Marullo *et al.* 2013). In contrast, other studies suggest that superoxide anions confer protective effects against H_2_O_2_-induced stress (Thorpe *et al.* 2013). The higher basal levels of superoxide anion in *eco1* mutant cells may explain PQ hypersensitivity of *eco1* mutant cells, compared with *mcd1* mutant cells.

A failure to modulate the cellular redox state in cells with reduced ESCO2/Eco1 function may expose an Achilles heel in both RBS and cancer. For example, RBS (a.k.a. pseudothalidomide syndrome) is phenocopied by the birth defects caused by exposure to the teratogen thalidomide (Herrmann and Opitz 1977; Waldenmaier *et al.* 1978; Sherer *et al.* 1991; Holden *et al.* 1992). ROS-neutralizing chemicals rescue teratogenic birth defects in the offspring of thalidomide-treated pregnant rabbits by reducing oxidative DNA damage (Parman *et al.* 1999; Wani *et al.* 2017). Moreover, genetic modulation of superoxide defense systems in *Drosophila* oocytes harboring cohesin mutations influences the rate of chromosome mis-segregation errors (Perkins *et al.* 2016, 2019). These results raise the possibility that increasing antioxidant levels may represent a treatment option to ameliorate the severity of birth defects otherwise present in RBS individuals. Separately, synthetic lethal approaches, through inhibition of either poly-ADP-ribose polymerase (PARP) or Wnt signaling, are effective treatments for tumors that exhibit marked changes in ESCO2-dependent pathways (McLellan *et al.* 2012; reviewed in O’Neil *et al.* 2013; Liu *et al.* 2018; Mondal *et al.* 2019; Waldman 2020; Chin *et al.* 2020). This raises the possibility that oxidative stress pathways may serve as an additional synthetic lethality target in treating tumors with mutated ESCO2 or cohesin.

## Data availability

Strains are available upon request. The authors affirm that all data necessary for confirming the conclusions of the article are present within the article. A strain table (Supplemental Table 1), figures, and tables with raw and complete images are available at figshare (https://figshare.com/authors/Robert_Skibbens/8138124).

## Supplementary Material

jkab426_Supplementary_Data
