# Correction to: Genetically induced redox stress occurs in a yeast model for Roberts syndrome

**DOI:** 10.1093/g3journal/jkac171

**Published:** 2022-07-20

This is a correction to: Michael G Mfarej, Robert V Skibbens, Genetically induced redox stress occurs in a yeast model for Roberts syndrome, G3 Genes|Genomes|Genetics, Volume 12, Issue 2, February 2022, jkab426, https://doi.org/10.1093/g3journal/jkab426

In the article by M. Mfarej and R. Skibbens entitled “Genetically induced redox stress occurs in a yeast model for Roberts syndrome”, Supplemental Figure 1 had a strain incorrectly listed as *smc3-159*, which has now been corrected to *smc3-42* (a ‘159’ allele of *SMC3* either does not exist or remains unpublished). The figure has been updated and the associated figure legend that previously read “A-B) 10-fold serial dilutions of *MCD1, mcd1-1, smc3-42, SCC2, scc2-4* mutant strains at the indicated conditions” now reads “A-B) 10-fold serial dilutions of *MCD1, mcd1-1, smc3-42, SCC2, scc2-4* mutant strains at the indicated conditions.”

In addition, a strain table (Supplemental Table 1) had not been published previously but is now available at figshare and can be retrieved from: https://figshare.com/articles/figure/Supplemental_Table_1_docx/20043524. Part of the Data availability previously read: “Figures, and tables with raw and complete images are available at figshare (https://figshare.com/ authors/Robert_Skibbens/8138124).” and now reads “A strain table (Supplemental Table 1), figures, and tables with raw and complete images are available at figshare (https://figshare.com/ authors/Robert_Skibbens/8138124).”

