# Supplementary material for: Genetically induced redox stress occurs in a yeast model for Roberts syndrome
Source: G3 (Bethesda). 2021 Dec 13;12(2):jkab426. doi: 10.1093/g3journal/jkab426 (PMC9210317; doi:10.1093/g3journal/jkab426)
Supplement: jkab426_Supplementary_Data [file jkab426_supplementary_data.zip › jkab426_Supplementary_Data/Suppl/jkab426_Supplementary_Data.docx]

**Figure S1**. Cohesion regulators promote cell growth in response to redox stress. A-B) 10-fold serial dilutions of *MCD1, mcd1-1, smc3-159, SCC2, scc2-4* mutant strains at the indicated conditions. In B, the 30˚C YPD control plate in the second panel on the left was reproduced in the first panel on the right side of the figure. Cells seeded on drug-treated plates were tested at a single semi-permissive temperature as indicated. Representative results shown from a total of N>2 biological replicates. Plates were imaged after two days of growth at the indicated temperatures.

**Figure S2**. Eco1 and cohesin promote cell viability in response to oxidative stress. Quantification of *ECO1*, *eco1W216G*, *MCD1*, and *mcd1-1* strain viability at 2-4 days following exposure to the indicated concentrations of H_2_O_2_ for 1hr at 30˚C. Percent viability was determined by averaging the ratios of viability (drug-treated colonies /untreated colonies) across N=6 biological replicates. Error bars indicate standard error of the mean. Statistical analysis was performed using two-tailed, Student’s T-test (alpha >0.05).

Figure S3. Experimental variability in fluorescence microscopy analyses of ROS levels in cohesion mutants. The composite data from all biological replicates of Figure 3 is shown where N>3. Each color on the plot represents a separate biological replicate. Dots of the same color correspond to a separate image taken within that biological replicate representing the average cellular integrated fluorescence density derived from all cells within that field of view. The black horizontal bars indicate the mean of all average cellular integrated fluorescence density values from those biological replicates. Error bars indicate standard deviation. A) H_2_DCFDA labeling in the indicated strains taken from untreated cells. B) DHE labeling in the indicated strains taken from untreated cells.

Figure S4. Flow cytometry analyses of H_2_DCFDA labeling in ECO1 and *eco1 W216G* cells. A-C) Three separate biological replicates representing H_2_DCFDA fluorescence levels in either untreated or H_2_O_2_-treated cultures of *ECO1* and *eco1 W216G* strains. Cells incubated within no dye were analyzed to establish background levels of FITC fluorescence in each strain. Then, untreated and H_2_O_2_-treated cultures were analyzed using the same parameters as the respective no dye control samples from that biological replicate. Any peak volume that is broader than the no dye control represents H2DCFDA signal generated from intracellular ROS detection.

**Figure S5.** Eco1 and cohesin mutation causes endogenous ROS overproduction following oxidative damage. A&D) Fluorescence micrographs of *ECO1, eco1 W216G, MCD1,* and *mcd1-1* strains that were treated for 1hr at 30˚C with 1mM H_2_O_2_. Following incubation at 30˚C for 1hr, cells were washed twice with PBS and incubated in either H_2_DCFDA or DHE for 2hrs at 23˚C prior to imaging. B&E) Quantifications of the data in A&C, respectively. Biological replicates N>4. Error bars indicate standard error of the mean. Statistical analysis was performed using one-tailed, Student’s T-test (alpha <0.05) for H_2_DCFDA staining and two-tailed Student’s T-test (alpha<0.05) for DHE staining. C&F) Experimental variability across all biological replicates of ROS labeling analyses in cohesion mutant cells treated with H_2_O_2_ wherein C corresponds to the data in A&B and F corresponds to the data in D&E. N>3. Each color on the plot represents a separate biological replicate. Dots of the same color correspond to a separate image taken within that biological replicate representing the average cellular integrated fluorescence density derived from all cells within that field of view. The black horizontal bars indicate the mean of all average cellular integrated fluorescence density values from those biological replicates. Error bars indicate standard deviation.
