# Supplementary figures and images for: Genetically induced redox stress occurs in a yeast model for Roberts syndrome
Source: G3 (Bethesda). 2021 Dec 13;12(2):jkab426. doi: 10.1093/g3journal/jkab426 (PMC9210317; doi:10.1093/g3journal/jkab426)

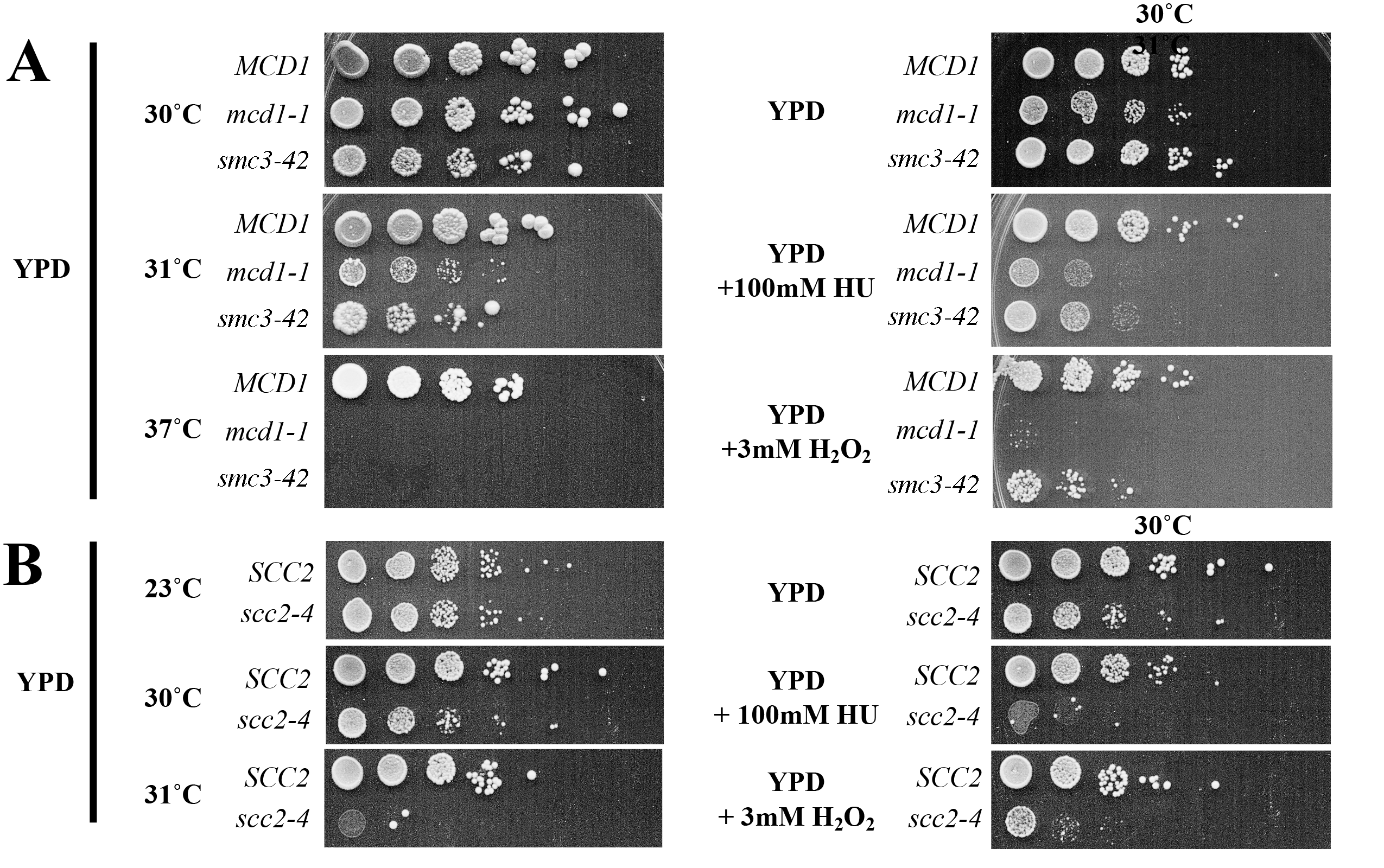

Supplement: jkab426_Supplementary_Data [file jkab426_supplementary_data.zip › jkab426_Supplementary_Data/Suppl/FigS1(corrected) (1).tif]

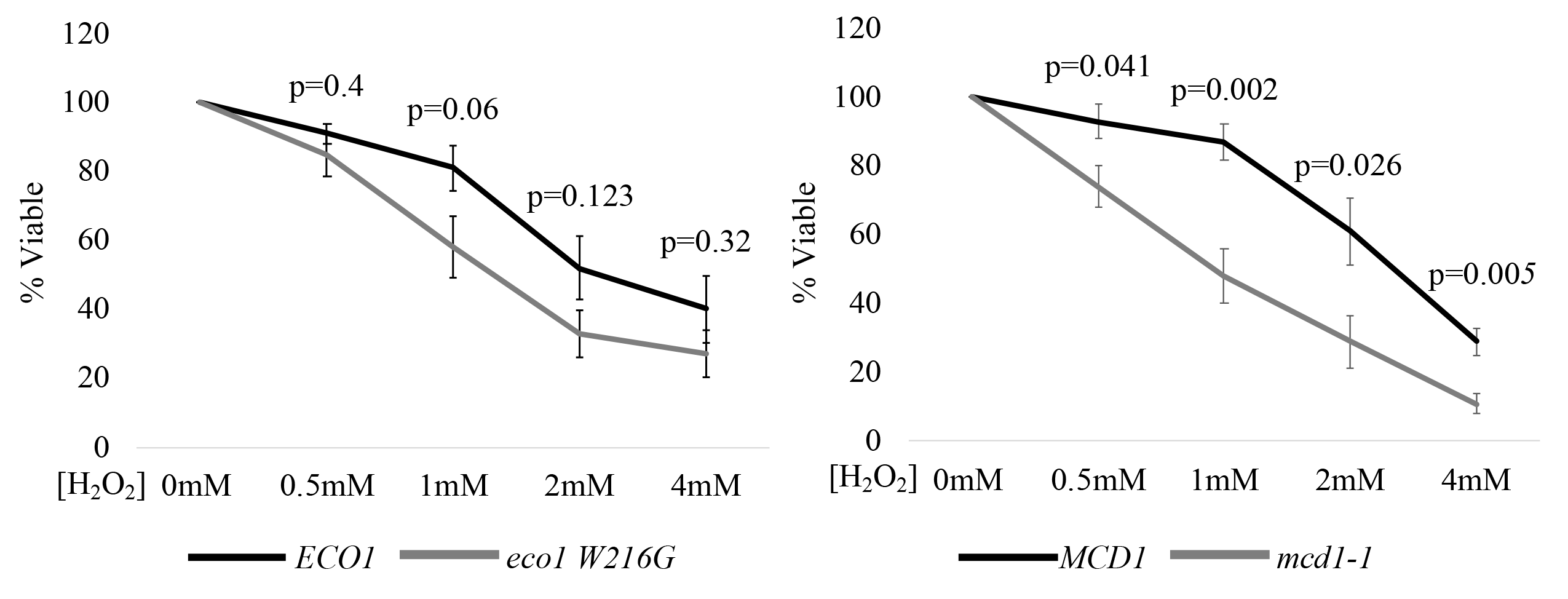

Supplement: jkab426_Supplementary_Data [file jkab426_supplementary_data.zip › jkab426_Supplementary_Data/Suppl/Figure_S2.tif]

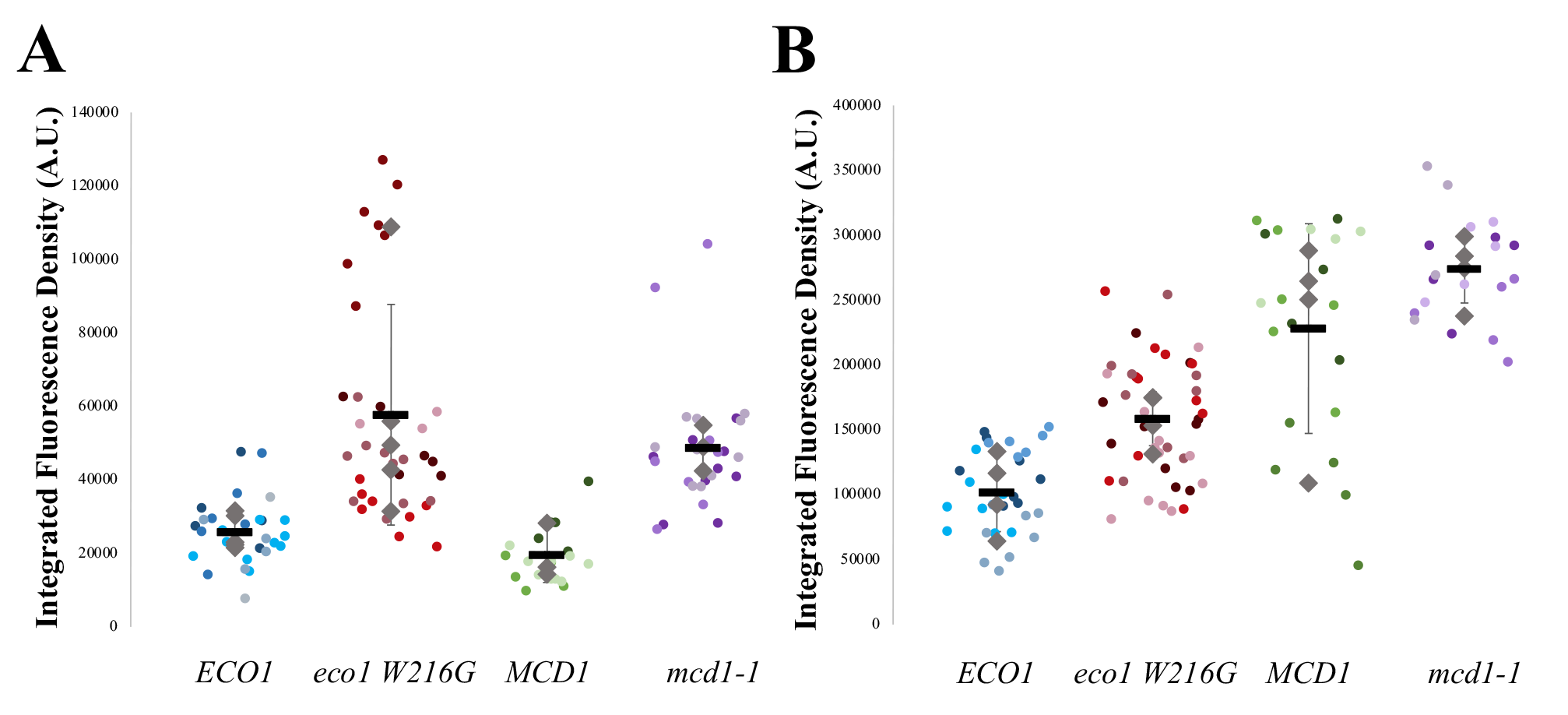

Supplement: jkab426_Supplementary_Data [file jkab426_supplementary_data.zip › jkab426_Supplementary_Data/Suppl/Figure_S3.tif]

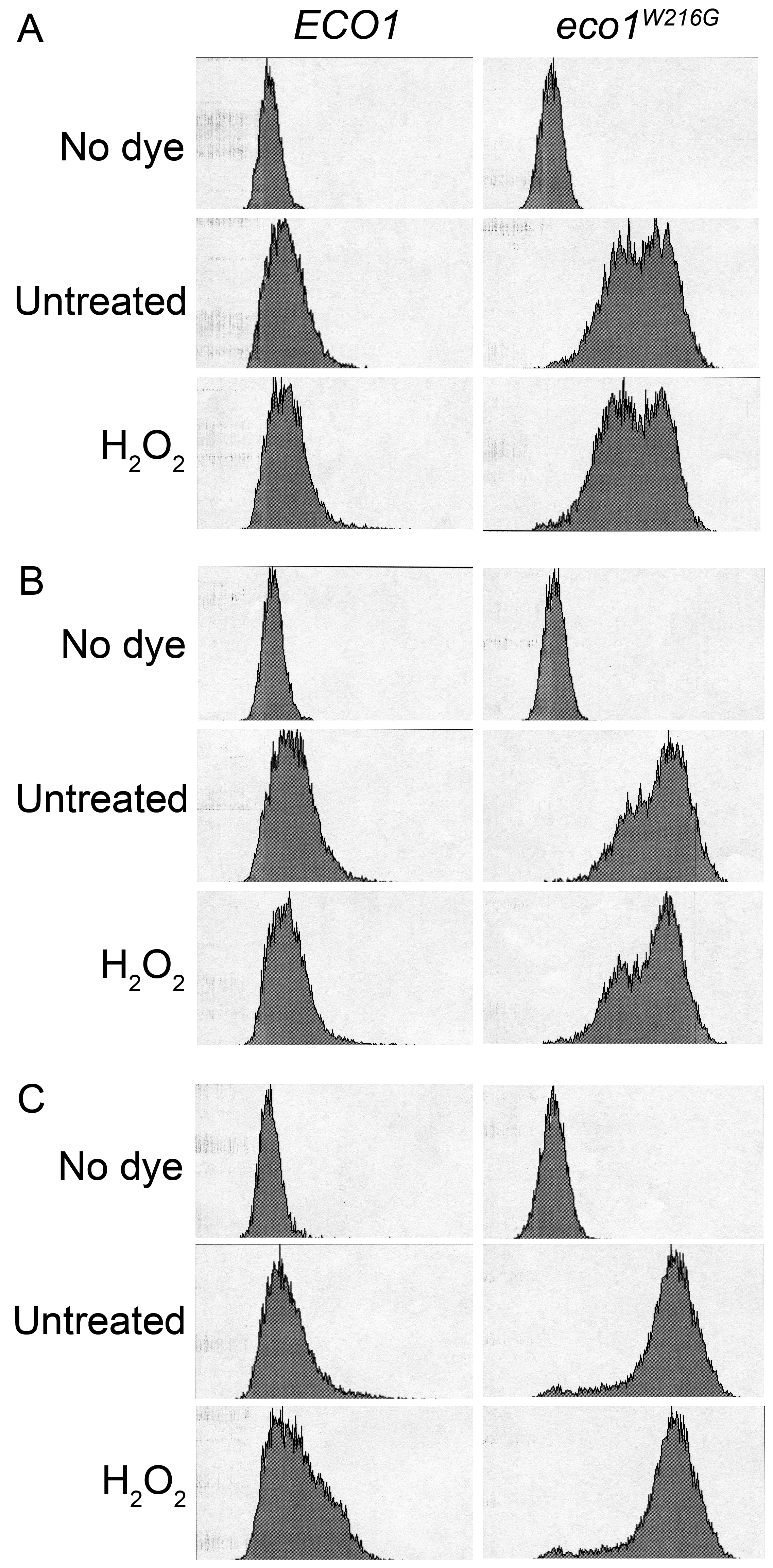

Supplement: jkab426_Supplementary_Data [file jkab426_supplementary_data.zip › jkab426_Supplementary_Data/Suppl/Figure_S4.tif]

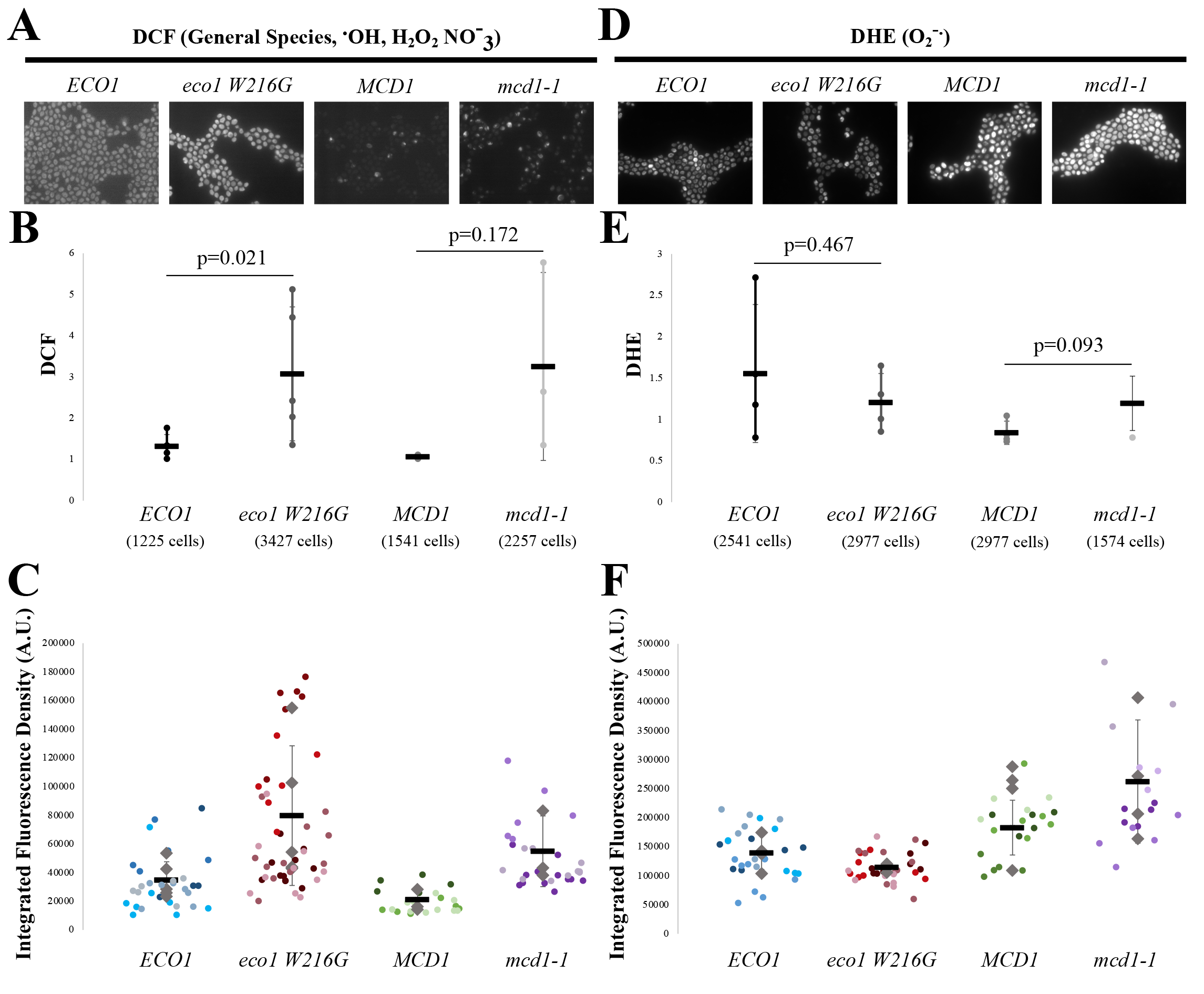

Supplement: jkab426_Supplementary_Data [file jkab426_supplementary_data.zip › jkab426_Supplementary_Data/Suppl/Figure_S5.tif]
